# Clinician and Patient Experience of Internet-Mediated Eye Movement Desensitisation and Reprocessing Therapy

**DOI:** 10.1007/s40737-022-00260-0

**Published:** 2022-02-03

**Authors:** Matthew Bursnall, Benjamin D. Thomas, Hannah Berntsson, Emily Strong, Mark Brayne, Daniel Hind

**Affiliations:** 1 School of Health and Related Research (ScHARR), University of Sheffield. Regent Court, 30 Regent Street, Sheffield, S1 4DA UK; 2 1a Havelock Road, Sheringham, NR26 8QD UK

**Keywords:** COVID-19, EMDR, Eye movement desensitization and reprocessing, Psychotherapy, Telehealth

## Abstract

Many eye movement desensitization and reprocessing (EMDR) therapists moved their practice online during COVID-19. We conducted surveys and interviews to understand the implementation and acceptability of online EMDR therapy. From 17 June to 2nd August 2021 an online survey was open to EMDR therapists from the EMDR Association UK & Ireland and EMDR International Association email lists, and, through them, their clients. Questions related to determinants of implementation (for therapists) and acceptability (for clients) of online EMDR. Semi-structured interviews were conducted with a sample of therapist respondents to provide a deeper understanding of survey responses. Survey responses were received from therapists (n = 562) from five continents, and their clients (n = 148). 88% of clients responded as being extremely or very comfortable receiving EMDR therapy online. At the initial point of ‘social distancing’, 54% of therapists indicated strong or partial reluctance to deliver online EMDR therapy compared to 11% just over one year later. Four fifths of therapists intended to continue offering online therapy after restrictions were lifted. Free-text responses and interview data showed that deprivation and clinical severity could lead to exclusion from online EMDR. Internet connectivity could disrupt sessions, lead to cancellations, or affect the therapy process. Therapists benefited from training in online working. Online EMDR is generally acceptable to therapists and clients, with reservations about digital exclusion, case severity, poor internet connectivity and the need for training. Further research is needed to confirm that online EMDR is clinically non-inferior to in-person working.

**Supplementary Information:**

The online version contains supplementary material available at 10.1007/s40737-022-00260-0.

## Background

Eye Movement Desensitisation and Reprocessing (EMDR) is a distinctive form of psychotherapy for stress-related conditions and a range of psychological health problems, which is clinically effective when delivered in-person [1]. It typically follows an eight-phase process: history taking,preparation; assessment; desensitisation; installation; body scan; closure; and, reevaluation [2]. It involves the use of bilateral sensory stimulation traditionally administered to the client by the therapist in-person, using eye movements, tones or physical taps [3–6]. During 2020, efforts to slow the spread of the Severe Acute Respiratory Syndrome CoronaVirus 2 (SARS-CoV-2, or ‘COVID-19’) meant that people in many countries were asked, or obliged, to stay at and therefore work from home. As a result, despite little prior training for online working, there was a high take-up of online consultation by mental health professionals [7]. EMDR Associations around the world rapidly drew up guidelines for using EMDR therapy online [8, 9], EMDR [10, 11]. One small, uncontrolled study suggested that effectiveness of EMDR therapy was unaffected by online delivery [12], but there was initial resistance from some professional bodies (e.g. EMDR Europe [10]. We conducted a survey of EMDR clients and therapists, supplemented by semi-structured interviews with therapists, to understand how the acceptability of EMDR therapy might be enhanced or disrupted by online delivery.

## Methods

The study consisted of cross-sectional online surveys of therapists and clients, followed by semi-structured interviews with a sample of therapists. Surveys were designed in collaboration with therapists to elicit views on the implementation, and client views on the acceptability, of online EMDR. The survey was pilot-tested by three therapists not involved in the initial design, resulting in six sections with 50 questions (Supplementary File). A client survey had 28 questions in ten shorter sections. The surveys employed multiple choice questions (~ 20%), Likert scales (~ 40%), free text (~ 30%) and ‘pick, group and rank’ tables (~ 10%), with three or five levels but a small number of questions. Both surveys were conducted using Qualtrics Research Suite (Qualtrics, Provo, UT), with a response window from 17/06/21 to 02/08/21. Ethical approvals did not permit collection of demographic information from the testing participants.

We surveyed all members of the EMDR Association UK & Ireland and EMDR International Association mailing lists, with a combined membership totalling > 8000 therapists in much of the English-speaking world, and—through them—their clients. Eligible clients were aged 18 or over, had received online EMDR, and were deemed by their therapists to be unlikely to find the survey challenging in terms of distress, comprehension or any other context-specific issue. No data on the characteristics of the target populations is available: assessment of the sample’s representativeness was not possible, and there was no adjustment for bias. The survey was not advertised; therapists were sent two reminder emails during the survey window. The chance of multiple participation was deemed low and not mitigated. Approaches to minimise human error were limited to blocking continuation until a participant ticked consent and double entry of email addresses for therapists consenting to contact.

To ensure confidentiality, demographics were limited to age group. Names were not collected, except where therapists consented to contact for the qualitative interviews. These data were stored on a secure file server accessible only to the University research team. Descriptive statistics were produced using STATA (StataCorp, College Station, Tx). There was no retention of partially completed surveys. The report includes the number of missing items for each question in the completed surveys. Item non-response ranged between 0 and 10%. The results have not been weighted to account for missing data.

EMDR therapists responding to the survey were invited to register interest in semi-structured interviews, of which a purposive sample of those who did, and did not, have bad experiences of delivering EMDR online was selected and approached by email. Six interviews are sometimes enough to achieve saturation [13, 14], small numbers are needed to satisfy focused aims using established theoretical frameworks [15]. We closed recruitment after nine interviews, the final three without substantially new thematic content [16]. Semi-structured interviews took place using a secure internet application, Google Meet, with an online consent procedure. An interview guide was developed in consultation with EMDR therapists (Supplementary file). Encrypted digital recordings were transcribed verbatim. Two researchers analysed each transcript and all free-text survey responses using NVivo Version 12 (QSR International), and the National Centre for Social Care 'Framework' analysis [16, 17] approach: familiarisation; identifying a thematic framework; indexing; charting; and, mapping and interpretation. A priori* coding* was to the Theoretical Domains Framework (TDF) [18] for therapist feedback on determinants of the implementation of online EMDR, and six out of seven constructs from Sekhon’s framework for client feedback on its acceptability [19].

### Ethical Considerations

This project received a favourable opinion from ScHARR Research Ethics Committee (037717) before commencement.

## Results

### Quantitative Findings

#### Survey Response

We received 562 therapist responses and 148 client responses, from the UK and Ireland (89%), North America (6%) and from elsewhere in the world (5%). A majority (66%) of therapists worked with adults only; 34% worked also with children and adolescents. All experience levels (years since basic EMDR training) and client types (public or private) were well represented. Most (84%) clients who responded were aged 25 to 65. Eighty-two percent had received 70% or more of their EMDR therapy online rather than in-person. There was an even split between people whose most recent programme of therapy was complete and those for whom it was continuing.

#### General Feelings

The majority of clients (93%) would be “very enthusiastic” (78%) or “fairly enthusiastic” (15%) about recommending online EMDR, with 88% feeling extremely or very comfortable receiving EMDR therapy online.

Sixty-two (42%) responding clients had experience of both online and in-person EMDR. Of these, 47 (76%) initially felt somewhat, very or extremely apprehensive about the switch to online EMDR. Once having experienced online EMDR, 34 (72%) of these felt extremely or somewhat comfortable and seven (15%) continued to feel somewhat or extremely uncomfortable. Overall, including all clients who responded, the majority (88%) felt extremely or somewhat comfortable with online EMDR. Whether or not they had experienced in-person therapy, all participants were asked for reasons why they felt EMDR might be enhanced when delivered online compared to in-person. The reasons most commonly given were that the respondent “felt secure in my own environment” (69%), “appreciated not needing to travel to my therapist’s place of work” (68%) and “found having my therapist’s face on screen to be reassuring and relationally connected”. The reasons most commonly given for why EMDR therapy might be compromised when delivered online compared to in-person were “poor internet connection” (35%), “distractions in/near the space I was using” (34%) and “difficulty interpreting body language” (20%).

When asked if they were initially reluctant to provide EMDR online, 54% of therapists strongly or partially agreed. By contrast, 11% strongly or partially disagreed with the statement that after a year working online,  they were now comfortable with providing EMDR online. In addition, they mostly felt safe working online (63% strongly and 24% partially agreeing) and were mostly comfortable about client confidentiality (67% strongly and 23% partially agreeing). A small minority, around 10% in each case, remained uncomfortable with delivering EMDR online and with safety and client confidentiality. Perhaps correspondingly, five per cent indicated they would revert as soon as possible to in-person only. Twenty-one percent expected to continue working almost exclusively online. The remaining 74% were intending to offer both ways of working, split evenly between preference for online or in-person.

Therapists felt either very bold (34%) or somewhat bold (44%) in taking action to make changes to fit the online environment. The number who did not deliver any EMDR therapy sessions online fell from 48% before the pandemic to around 13% during the first lockdown period, and continued to drop subsequently. The therapists gave very similar enhancing and compromising factors to those identified by clients. The reasons most commonly given for enhancement were “I appreciated not needing to travel to a separate place” (70%), “feel secure in my own environment (58%) and “I feel this way of working is more contained and focused” (40%). The compromising reasons most commonly given were poor internet connection (77%), difficulty interpreting body language (44%) and poor video quality (37%).

#### Platforms/Security Issues

Most clients said they initially met on Zoom (64%) or Bilateral Base (14%). That stayed largely stable with time, with Zoom at 60% and Bilateral Base increasing slightly to 22%. Similarly, 54% of therapists preferred Zoom, with 18% preferring Microsoft Teams or Attend Anywhere (nine per cent each), and eight per cent choosing Bilateral Base. A further 18% said they used Zoom sometimes, with a further 10% and nine % respectively also frequently using Skype and MS Teams.

Amongst therapists, 337 (62%) were initially somewhat troubled (55%) or very troubled (7%) by online confidentiality, and by ethical approval and security issues. Of these, after a year of working online, 133 therapists remained somewhat troubled (39%) but 194 were now “not troubled at all” (55%). Overall, 75% were now not troubled at all, 24% were somewhat troubled and just one percent were very troubled (compared to the seven percent who were initially very troubled).

#### Method of Bilateral Stimulation

Butterfly taps were the method of bilateral stimulation (BLS) most commonly (43%) identified by clients experiencing EMDR online, followed by online eye movement (16%), dots on either side of the client’s screen (9%) or bilateral tones generated at the client end (7%). Hand movements on screen were reported by seven percent of clients.

Butterfly taps were identified as the preferred method of 54% of therapists, and also used by a further 18% of therapists alongside other forms of BLS.

Among therapists, 140 (25%) said they had switched their method of generating bilateral stimulation since first working online. The most common switch was from online eye movements using therapists’ own traditional arm movements to butterfly taps (3.2%), butterfly taps to screen side dots at the client end (3.4%)—consistent with client preferences mentioned above—and between butterfly taps and other online arm movements and vice-versa (2.3% and 2.5% respectively). Overall, the switching pattern suggests therapists and clients have been experimenting effectively until they find the online approach that works best for them.

#### Dealing with Issues

A strong majority of therapists (88%) found that building relationships with clients was extremely or very effective online. A slightly lower proportion (72%) reported dealing effectively online with intense client affect and abreactions (69%). An issue handled less effectively online was felt to be disassociation (52% reported handling it extremely or very effectively, 38% only moderately).

Clients were invited to comment on the effectiveness of seven aspects of online sessions: “Bilateral Stimulation”; “Installation of a safe/special place and resources”; “Focus on specific memory targets”; “Identifying negative thoughts, emotions and body sensations before sets of BLS”; “Identifying an alternative positive cognition/thought before starting processing”; “Use of numerical scales to check progress—subjective units of disturbance (SUDs) and validity of cognition (VoC)”; “Tight session structure with regular appropriate returns to the target memory”. More than two thirds of clients found the first four (BLS, special place, alternative PC, session structure) to be extremely or very effective online. The remaining three (specific targets, NCs and body sensations) had marginally less positive responses, with 44% and 48% finding them slightly effective.

#### Interview Data (n = 9)

Therapist interviews were conducted with a sample of the survey respondents from the UK, US, Romania, India, South Africa and New Zealand (Table 1). Many therapists reported surprise at how effective online EMDR had been, some believing it had improved them as a therapist (Table 2: *Optimism*). A number reported fear as a barrier to initial take-up of online EMDR; after starting to work this way, they could become demotivated by technical issues and the fatigue associated with online working (*Emotion*). Therapists were motivated to work online by a sense of responsibility for the unmet need of clients. Some were concerned that, compared with in-person work, online EMDR could compromise the therapeutic connection, or they had safety concerns (*Social or Professional Role*). In particular, some reported that seeing only the head and shoulders restricted their ability to read nonverbal cues; others were concerned they would be unable to deal with abreactions, because they were not co-present or because of internet connectivity issues (*Beliefs about capabilities*). As a result, some reported watering down therapy, concentrating on resourcing but not processing (*Intentions*). Nonetheless, therapists commonly believed that clients benefited from being in their own space, which added to their sense of safety, made them more willing to access and process traumatic experiences, and allowed them to take greater control of their recovery. In this regard a greater emphasis on resourcing was cited by some therapists: Western therapists cited the use of pets, treasured objects and imagery associated with beneficent individuals, whereas one Indian therapist flagged the use of processes, such as prayer and yoga breathing. Therapists also reported fewer cancellations (*Beliefs about consequences*). Relief from commuting and the ability to deliver more sessions during the day provided an incentive for some therapists to work online (*Reinforcement*). Therapists expressed a variety of goals with some wishing to move wholly online in the future, others looking forward to mainly working in-person again, and others still intending to incorporate lessons of the pandemic period into future practice (*Goals*).

**Table 1:** Participant characteristics

	Therapist survey (N = 562)	Interviews (N = 9)
*Location*		
UK and Ireland	499 (89%)	4
North America	34 (6%)	1
Australia/NZ	15 (3%)	1
Europe	6 (1%)	1
Rest of World	8 (1%)	2
*Occupation*		
Psychotherapist	228 (41%)	3
Psychologist	147 (26%)	5
Counsellor	90 (16%)	1
Cog. behav. therapist	52 (9%)	–
Nurse	16 (3%)	–
Psychiatrist	5 (1%)	–
Other	24 (4%)	–
*Time since basic EMDR training*		
< 1 year	45 (8%)	–
1 to 4 years	189 (34%)	5
5 to 10 years	186 (33%)	3
More than 10 years	142 (25%)	1

**Table 2:** Therapist feedback, classified using the Theoretical Domains Framework

Construct	Illustrative quotations
***Psychological capability***	
*Skills* Knowing “how to” deliver therapy online, including training	*Barriers* “I’m not able to see any cases because I don’t know how to use it online… then I realised there were a bunch of us that didn’t know how to do it, so there was training for that” “I know there’s a whole heap of technology that can take care of that, but I didn’t because…I’m not a techno whiz at all” *Facilitators* “Lots of Zoom training to develop myself, lots of sharing and communities of practitioners talking” “…the…EMDR Association… did some workshops with us…and they helped us with the online err techniques…so we had their help and err this was good”
*Knowledge* Knowledge about online EMDR	“I would say that in the last 15 months there’s been a significant number of articles that have appeared that demonstrate that both individual and group-administered EMDR therapy can be provided remotely, effectively, safely. That there are a number of protocols that you can follow to make sure that it’s safe and appropriate to offer EMDR. And the research suggests that it can be just as effective as delivering EMDR in person” “ So, how are we going to do this online? In the office I tend to use a lightbar or I use tappers for the…bilateral stimulation. And so “How am I gonna do that online?” So, that was a concern” “..I have worked out how, I know how to, I don’t worry about the eye movement stuff”
*Memory, attention and decision processes* The ability to focus selectively on aspects of the environment and choose between two or more alternatives	“The other thing that was really different about my EMDR work once I was working online is that prior to doing any processing, I asked my clients to gather a resource kit around them” “I am probably more cautious about ensuring stability and resourcing before processing” “…to do the eye movement…on the screen…I just think…it’s difficult because you don’t know how the client is perceiving this on their side and maybe if it’s a bad connection…you don’t know if the screen is wide open or it’s just a little block…and if you don’t get that full eye movement, it’s not really bilateral stimulation—and with this butterfly hug, it’s straightforward…your screen can be small, it can be wide, you can’t miss this”
*Behavioural regulation* Managing objectively observed actions/ monitoring/ action planning	“…we just stuck to knee taps and I quite like knee taps because I can hear it…or you can see it because you can see the hands going so…you’ve got a sense of how fast they’re going and when they’ve stopped…” “I talked a lot with the clients about leaving the therapy space and going straight into family life. And that was on the basis of my own experience of that happening. I’d leave my therapy room and my grandchildren would be demanding me to do something, before I’d really stopped being a therapist. And so, I was explaining to clients – “What are you going to do when you finish the therapy before you go back into being mum?”…normally they’d have a drive home. They might go and have a coffee, they might sit in the car in the car park, or they would have more time in that transition. When you’re online, you switch the computer off and there’s no transition time” “…I would never have dinner sat here now…I will sit in another space either across here or the chair across here because this, to me, this is my working space…and if I were to have EMDR trauma therapy in this space the next time I sat here for dinner I might be like oh all those associations to the trauma memories kind of thing so you want to maybe have a space that’s set aside”
***Opportunity***	
*Environmental context and resources* Situations that discourage or encourage the implementation of EMDR online (environmental stressors/ resources/ organisational culture)	“Although I have excellent internet connection that is not always true for my clients” “…poorer patients usually have poorer devices and weaker internet connection…” “I feel more confident working online because I can have different screens open i.e. the protocol, Negative beliefs/positive beliefs/interweaves. If this was F2F I would have pages everywhere! It feels more slick online for me” “For me, a therapeutic relationship is when I’m talking to the client and giving them my full attention. I absolutely was not really comfortable with having to hold a book in my hand and to read from it. So, that was one thing…it doesn't look like you’re reading from a script…You get the assumption that “Probably this one doesn’t know what she’s talking about if she has to look at a book” “Access to clients increases with online. More people can therefore get the benefit of EMDR”
*Social influences* *Interpersonal processes that facilitate or hinder the implementation of EMDR online*	“Privacy for the client is not always guaranteed—on one occasion, despite me having asked whether the client was on her own, her partner was actually in the room out of camera sight” “…her mother was one of the things that we needed to talk about and the fact that her mother might simply be downstairs or going to the loo upstairs creates a presence, which I think, messes up the EMDR…so that sense of clients not having a confidential space is a killer” “We’re hoping that nobody rings a bell, you’re hoping that nobody crosses the room while you’re having the session…I have teenage daughters, so I’m just hoping really that they don’t fight in the middle of the session”
***Motivation***	
*Social or professional role and identity* *A declared coherent set of values in the work setting (compatibility of online working with professional role/identity/ standards/)*	“The main thing in therapy in general is you, you need to connect with your client, and this is the challenge in doing it on an online platform” “The risk to the clients is they get further into an abreaction before I notice the need to bring them out of it. And because I’m not there, it’s harder for me to bring them out of it…” “…my fear of the beginning of…lockdown, was that I wouldn’t be able to offer EMDR online…I wondered what that would be like, if that sort of opportunity wasn’t there to offer clients as part of a treatment agreement” “ I’m definitely very choosy about selecting my, taking them up with the EMDR for online sessions”
*Beliefs about capabilities* Perceived competence/ confidence in ability to deliver therapy online (ease/difficulty of online delivery)	“…the number of pixels on the screen is just not nearly enough for me to see the visual details that I am accustomed to, as well as the rest of your body being off screen. And so, I feel like I’m missing huge portions of the information that I need. It’s like, you know, doing EMDR in the dark kind of thing” “I like having lots of non-verbal cues. I like seeing what their body anxiety is doing, as well as their head and shoulders. Head and Shoulders is a shampoo for me. It’s not a therapy mode…I like the whole person in the space” “It makes me double cautious and triple cautious because I don’t want to activate, you know, open the proverbial can of worms if I’m not able to go and pick them up…And when the connection is not reliable, I don’t know that halfway through the activation I might be leaving my client stranded with a can of worms half open and everything crawling everywhere, and I can’t pick it up” “I think once I’d got my head around the idea that it’s possible to be online with your clients, it’s possible to have an emotional connection, to have a sense of containing them, you’re contained, they’re contained and have trust in the therapeutic relationship online”
*Optimism* Confidence that things will happen for the best/ desired goals will be attained	“I have experienced that it is equally effective and rewarding, and no change in the relationship, therefore I appreciate the convenience of working from home” “Been totally surprised at how effective working online has been” “I think it’s err err umm (tuts) – what would you say, the evolution of (laughs), of therapy” “I believe that it has made me a better EMDR therapist” “It has increased my flexibility of approach and creativity in using EMDR”
*Beliefs about consequences* Beliefs about the outcomes of online therapy	“Some clients appear to benefit from being in their own space at home which adds to their sense of safety and can facilitate willingness to access and process traumatic material” “In terms of them taking more responsibility for resourcing themselves at the start, I think that’s a brilliant model, because then they’re becoming more self-reliant, more self-sufficient, less dependent on the therapist and so more engaged in their own recovery, taking an agency and responsibility for their own recovery” “I help them work it out on their own without me being there and that sense of independence and that resource and I’ve really seen that it actually works faster and much better, because to start with I am not handholding them to that extent that we generally do in a face-to-face session” “I have had less drop outs and better attendance so better in terms of mostly always completing the work”
*Intentions* A conscious decision to perform a behaviour or resolve to act a certain way, in response to/during online therapy	“I just don’t get the can of worms open, but do damage control, do anxiety release, do resourcing, and it does help, you know? Clients, at least they don’t get worse during that time. They haven’t really gotten any better, it’s kind of keeping a stalemate situation and they didn’t get as bad as could have been without that support” “Therefore, I water it down a lot and I don’t actually do what I call therapy. We do prevention, we do damage control” “Uh, initially, I decided not to do it. Not to do processing online. And that decision was probably my own fear about whether I could contain people well enough online”
*Goals* Outcomes that an individual wants to achieve	“I would encourage them to bring their own things from home that they then take home again. So, that would be a change that I would consider continuing after” “I actually want to work only online…I’m not going back to the office, it was so good for me”
*Reinforcement* Incentives/rewards for moving to online therapy	“the online sessions have been better because it impacts on the travelling time and it saves a lot of time in the day. Still mentally fresh from that point of view” “…so at the beginning, I could work, because I work with trauma just with five clients per day—this was my maximum. Because of the pandemic I got to work with sometimes more than 10 clients a day…”
*Emotion* Emotional responses (positive or negative) to online therapy	“…it’s quite scary to be potentially leaving people in dissociated states without having a way to pick up the pieces” “I’m petrified that I leave my clients in a painful situation and I couldn’t pick it up” “I get most frustrated by the issues with tech (not always internet connection—sometimes the EMDR apps are flakey)” “I am far more tired and strained online seeing 6 clients or couples each day on the screen as I have to focus really hard to pick up nuances in energy, subtle body changes, observations of facial colour changes to deal quickly with dissociation, abreactions, switching (for DID clients) and epileptic fits”

While most therapists admitted little initial understanding of whether online EMDR was appropriate, or which the tools could support it, most acknowledged developing in this regard over the lockdown period (*Knowledge*). Training workshops and webinars online developed the skills of some who expressed initial discomfort with technology (*Skills*). Therapists reported having to ensure stability and resourcing before processing, and to pay particular attention when delivering bilateral stimulation (*Memory, attention and decision processes*). For safety, they chose bilateral stimulation techniques that could be easily monitored; taking care how they and the client should interact with people immediately after sessions; and how the space used for therapy might be used/inhabited at other times (*Behavioural regulation*).

Internet connectivity was a concern, sometimes interrupting the flow of therapy sessions or interfering with the monitoring of EMDR procedures and client safety. Healthcare organisations often limited the range of software and other technologies which therapists could use, which could be frustrating where there was poor acceptability or reliability. Some therapists reported using telephone contact as a back-up when an internet connection failed. Therapists found that online working increased their client base beyond existing geographic limits and some found unexpected advantage in clients not being able to see them reading from scripts or referring to manuals (*Environmental context and resources*). Therapists often expressed concern that clients might have difficulties finding private space for online EMDR, especially when they were cohabiting with individuals associated with their trauma. Therapists were concerned that children, pets or delivery workers (“the man from Porlock” [20], as one therapist put it, in a literary allusion to the disturbance of creativity creativity), at either end of the connection, might interrupt therapy—although a surprising number of clients were reported to bring dogs into therapy, finding stroking their pets a stabilising experience (*Social influences*).

##### Client Feedback

That online EMDR offered continuation of therapy during lockdown and a sense of control from being in one’s own environment was of value to many (Table 3: *Ethicality*). Online EMDR removed the need for travel, allowing easier access and uninterrupted post-session reflection; but, it was subject to interruptions by dependents, callers and internet connectivity problems (*Burden*). Some therapists discussed how they and their clients sacrificed privacy to participate in online EMDR, giving up their home to be a space with difficult therapeutic associations; but, relieved of the need to travel, clients appreciated reallocating time to family, professional and leisure activities (*Opportunity cost*). Some clients appreciated the convenience and comfort of receiving therapy in their own home, feeling less inhibited about disclosure and therapeutic engagement. Others reported difficulty relaxing, engaging, or connecting with their therapist, citing the impersonality of the format (*Affective attitude*). Some, with prior experience of in-person EMDR, found online EMDR inferior; others found it difficult to establish a therapeutic relationship; others still found the online EMDR surprisingly effective, citing the experience as more immersive (*Perceived effectiveness*). Clients who had experienced in-person therapy sometimes discussed feeling more in control during online EMDR, but others worried about their *not* being in control during therapy. (*Self-efficacy*).

**Table 3:** Client feedback classified according to Sekhon’s acceptability framework

Construct	Illustrative quotations
*Affective attitude* How the individual feels about online EDMR	Positive *I felt less inhibited online and my triggers around closeness *etc.*were no longer there* *…being in your own home makes you feel more secure* Negative *I really missed the being in the same space as my therapist. It felt more impersonal online* *I feel a lot of my connection and healing is *via* physical human to human contact. This is not present online. I also prefer the therapist to be tapping me whilst I do the therapy, and I prefer this to the ear buzzers. I feel she is caring for me more in person, and I feel more connected to her in person*
*Burden* How much effort that the individual recognises is needed to engage in online EDMR	Online EDMR is a burden *It was tricky sometimes finding a place that I wouldn't be disturbed* *[…] was difficult with two children and barking dog to feel completely at ease* Online EDMR is not a burden *After processing it is easier to do self-care activities right after the session and relax in my home environment, especially after a difficult session where processing continues and you feel a bit hazy, it’s better than driving/ travelling home when you feel that you need time for yourself, you are already home* *‘The software used was very easy as the therapist managed it without me needing to worry*
*Ethicality* Is online EDMR deemed a good fit for the individual	*Having EMDR online made me feel like I was more in control of the process which is very important to me.*
*Opportunity costs* The extent to which the individual forgoes other aspects of their life to engage in online EDMR	*I feel that due to me using it to ease trauma I felt it allowed the work to spill further being that I was home, whereas previously I subconsciously left my emotional stuff in the therapist's room and in all senses, closed the door behind me!’*
*Perceived effectiveness* The extent to which the individual perceives online EDMR to be effective	Ineffective *I just did not get the same sort of experience working closely with my counsellor; face to face in a private safe room was so much more beneficial.’* *To be honest, I much prefer in-person with my therapist. I think seeing all of the person (both ways) is more beneficial. I do not mind working remotely, but it is just that. Remote* *If you start working with a therapist (remotely) on day one and then begin EMDR, you will lose the organic relationship needed for EMDR to be effective* Effective *Actually I liked online as much as in person. I was surprised it would work remotely but it did!’*
*Self-efficacy* The extent to which the individual is confident in their ability to engage in online EDMR	Ability to engage in online EDMR *I felt as if it was easier for me to be able to just stop the session at any point that I needed to just at the touch of a button. Knowing that I could end the session so easily and that everything was within my control made me feel far more relaxed and confident with the process* Reduced ability to engage in online EDMR *‘However, because I work online on video for a living I found it difficult to get into my emotions and not be in 'work mode' as I associate my home office with work related activities and not emotional self exploration so I found it hard to connect with the feelings and allow myself to just 'be' online because I associate video calls with being an IT supervisor at work*

## Discussion

### Principle Findings

Internet-mediated EMDR delivered online is acceptable to around nine out of ten therapists and clients during a pandemic, with four fifths of therapists intending to offer this modality after restrictions are lifted.

### Strengths and Limitations

This is the largest EMDR-related survey of which we are aware. Our research was confined to anglophone countries, and respondents from the UK predominated; nonetheless, responses from five continents provide reassurance about the generalisability of our findings and offer insights into culturally specific responses. Client enthusiasm for online EMDR should be treated with caution as those more comfortable with an online environment are more ready to respond to internet surveys [21–23]. Clients with previous negative experiences of telehealth, those who have no access—or poor access—to the internet due to deprivation or rurality, and people with clinically severe conditions, may opt out, or be selected out, of online EMDR by therapists.

### Implications for Clinicians, Policy-Makers and Further Research

Online therapy in other areas of mental health is generally acceptable, if not universally so [24]. However, online consultations tend to be used by younger, more affluent, and educated groups, and may increase health inequalities [25]. Technical problems cause cancellation and rescheduling of telehealth consultations [26], and the absence of in-person contact may have negative consequences or adversely affect the physician–patient relationship [24], however, in a pandemic, the counterfactual is that similar or greater numbers of therapists would be concerned with the impacts of face masks on the quality of in-person therapy [27].

More generally, training for, and support of, therapists adopting online working is essential [7, 28], to address concerns about standards, legal aspects, online privacy, security and data storage [24]. Telehealth requires robust infrastructure in terms of hardware, network connections and technical support [24], which is challenging for independent providers. Widely-used interventions are not always better than alternatives [29], and randomised controlled trials are needed to confirm that EMDR therapy online is clinically non-inferior to in-person working [30].

## Supplementary Information

Below is the link to the electronic supplementary material.

Supplementary file1 (DOCX 33 kb)
