# Supplementary material for: Clinician and Patient Experience of Internet-Mediated Eye Movement Desensitisation and Reprocessing Therapy
Source: J Psychosoc Rehabil Ment Health. 2022 Feb 3;9(3):251–62. doi: 10.1007/s40737-022-00260-0 (PMC8812350; doi:10.1007/s40737-022-00260-0)
Supplement: Supplementary file 1 — Supplementary file1 (DOCX 33 kb) [file 40737_2022_260_MOESM1_ESM.docx]

SUPPLEMENTARY FILE: Full statistical report and interview schedule

**Therapist Tables**

Table S1 Therapist context

| Question | Response (only one) | n (%) |
| --- | --- | --- |
|  |  | (N=562) |
|  |  |  |
| What country do you live in | Australia/NZ | 15 (3%) |
|  | Europe | 6 (1%) |
|  | North America | 34 (6%) |
|  | Rest of World | 8 (1%) |
|  | UK & Ireland | 499 (89%) |
|  |  |  |
| How long ago did you do your basic EMDR training? | 1 to 4 years | 189 (34%) |
|  | 5 to 10 years | 186 (33%) |
|  | Less than one year ago | 45 (8%) |
|  | More than 10 years ago | 142 (25%) |
|  |  |  |
| How would you describe the type of therapist you are | Cognitive Behavioural Therapist | 52 (9%) |
|  | Counsellor | 90 (16%) |
|  | Nurse | 16 (3%) |
|  | Other | 24 (4%) |
|  | Psychiatrist | 5 (1%) |
|  | Psychologist | 147 (26%) |
|  | Psychotherapist | 228 (41%) |
|  |  |  |
| Which types of client do you work with | NHS (UK) / HCE (RoE) | 89 (16%) |
|  | NHS (UK) / HCE (RoE),Other (pl | 2 (0%) |
|  | Other (please specify in the b | 21 (4%) |
|  | Private | 222 (40%) |
|  | Private,NHS (UK) / HCE (RoE) | 106 (19%) |
|  | Private,NHS (UK) / HCE (RoE),O | 15 (3%) |
|  | Private,Other (please specify | 43 (8%) |
|  | Missing Data | 64 (11%) |
|  |  |  |
| Which age groups do you work with? | Adolescents,Younger children | 7 (1%) |
|  | Adults | 373 (66%) |
|  | Adults, Adolescents | 102 (18%) |
|  | Adults, Adolescents, Younger children | 76 (14%) |
|  | Adults, Younger children | 4 (1%) |
|  |  |  |

Table S2 Number of online sessions before and during pandemic

| Number online sessions before the March 2020 lockdown | 1 or 2 sessions per week | 63 (11%) |
| --- | --- | --- |
|  | 3 to 10 sessions per week | 101 (18%) |
|  | More than 10 sessions per week | 58 (10%) |
|  | No sessions | 270 (48%) |
|  | Occasional sessions | 67 (12%) |
|  | Missing Data | 3 (1%) |
|  |  |  |
| Number online sessions during the 1st lockdown March to Summer 2020 | 1 or 2 sessions per week | 116 (21%) |
|  | 3 to 10 sessions per week | 180 (32%) |
|  | More than 10 sessions per week | 121 (22%) |
|  | No sessions | 71 (13%) |
|  | Occasional sessions | 69 (12%) |
|  | Missing Data | 5 (1%) |
|  |  |  |
| Number online sessions in 2020 since Summer 2020. | 1 or 2 sessions per week | 109 (19%) |
|  | 3 to 10 sessions per week | 228 (41%) |
|  | More than 10 sessions per week | 154 (27%) |
|  | No sessions | 32 (6%) |
|  | Occasional sessions | 35 (6%) |
|  | Missing Data | 4 (1%) |
|  |  |  |
| Number online sessions currently since Jan 2021 | 1 or 2 sessions per week | 96 (17%) |
|  | 3 to 10 sessions per week | 248 (44%) |
|  | More than 10 sessions per week | 163 (29%) |
|  | No sessions | 18 (3%) |
|  | Occasional sessions | 33 (6%) |
|  | Missing Data | 4 (1%) |
|  |  |  |

Table S3 Proportion of clients unwilling or unable to work online

| Question | Response (only one) | n (%) |
| --- | --- | --- |
|  |  | (N=562) |
|  |  |  |
| Proportion of sessions online | <10% | 152 (27%) |
|  | 10% to 49% | 289 (51%) |
|  | 50% to 79% | 50 (9%) |
|  | >80% | 64 (11%) |
|  | Missing Data | 7 (1%) |
|  |  |  |

Table S4 General feelings about acceptability of online EDMR before pandemic and now

| I was initially reluctant to provide EMDR online | neither agree nor disagree | 80 (14%) |
| --- | --- | --- |
|  | partially agree | 197 (35%) |
|  | partially disagree | 57 (10%) |
|  | strongly agree | 117 (21%) |
|  | strongly disagree | 111 (20%) |
|  |  |  |
| I am comfortable with providing EMDR online | neither agree nor disagree | 10 (2%) |
|  | partially agree | 109 (19%) |
|  | partially disagree | 26 (5%) |
|  | strongly agree | 379 (67%) |
|  | strongly disagree | 33 (6%) |
|  | Missing Data | 5 (1%) |
|  |  |  |
| I feel safe working online | neither agree nor disagree | 30 (5%) |
|  | partially agree | 134 (24%) |
|  | partially disagree | 22 (4%) |
|  | strongly agree | 354 (63%) |
|  | strongly disagree | 19 (3%) |
|  | Missing Data | 3 (1%) |
|  |  |  |
| I am sufficiently comfortable about client confidentiality | neither agree nor disagree | 12 (2%) |
|  | partially agree | 132 (23%) |
|  | partially disagree | 15 (3%) |
|  | strongly agree | 377 (67%) |
|  | strongly disagree | 22 (4%) |
|  | Missing Data | 4 (1%) |
|  |  |  |
| How enthusiastic are you about providing online EMDR | I now expect to continue working almost exclusively online | 118 (21%) |
|  | I will encourage clients to work with me in-person but work online if that is their preference | 193 (34%) |
|  | I will encourage clients to work with me online, but work in-person if that is their preference | 222 (40%) |
|  | I will revert to working in-person only as soon as possible | 26 (5%) |
|  | Missing Data | 3 (1%) |
|  |  |  |

Table S5 Enhancing and Compromising Factors of online EMDR compared to in-person

|  | Method | n (%) |
| --- | --- | --- |
|  |  | (N=562) |
| Enhanced | Feel secure in my own environment e.g. home | 327 (58%) |
|  | Working via a screen helps me focus on the therapy | 193 (34%) |
|  | I appreciated not needing to travel to a separate location | 395 (70%) |
|  | Can tap-in client’s special place / use resource | 116 (21%) |
|  | I feel more secure/more at ease in establishing rapport | 71 (13%) |
|  | I feel this way of working is more contained and focused | 223 (40%) |
|  | Easier to process client material online. | 69 (12%) |
|  | I found the closeness reassuring and relationally connected | 151 (27%) |
|  | Easier to co-create a supportive space. | 172 (31%) |
|  | Other (Please elaborate below) | 149 (27%) |
|  |  |  |
| Compromise | Difficulty in finding a secure and confidential ph | 68 (12%) |
|  | Distractions from people in the same room/building | 92 (16%) |
|  | Privacy issues | 90 (16%) |
|  | Internet security issues | 93 (17%) |
|  | Difficulty facilitating emotion | 64 (11%) |
|  | Difficulty interpreting body language | 246 (44%) |
|  | Technical incompatibility | 161 (29%) |
|  | Poor internet connection | 430 (77%) |
|  | Poor audio quality | 177 (31%) |
|  | Poor video quality | 210 (37%) |

Table S6 Courage in making adaptations

| How bold do you feel yourself to have been in taking the risk of trying new ways of working with/using EMDR online | Neither bold nor timid | 98 (17%) |
| --- | --- | --- |
|  | Somewhat bold | 256 (46%) |
|  | Somewhat timid | 13 (2%) |
|  | Very bold | 190 (34%) |
|  | Very timid | 4 (1%) |
|  | Missing Data | 1 (0%) |
|  |  |  |

Table S7 Primary software platform

|  | Zoom | 302 (54%) |
| --- | --- | --- |
|  | Skype | 20 (4%) |
|  | Microsoft Teams | 53 (9%) |
|  | Bilateral Base | 45 (8%) |
|  | Google Meet | 5 (1%) |
|  | Attend Anywhere | 49 (9%) |
|  | VSee | 2 (0%) |
|  | WhatsApp | 7 (1%) |
|  | Facetime | 2 (0%) |
|  | Facebook Messenger | 1 (0%) |
|  | Telephone | 1 (0%) |
|  | Other | 59 (10%) |
|  | None given | 16 (3%) |
|  |  |  |

Table S8 Platform also used often

| Platform | n (%) |
| --- | --- |
|  | (N=562) |
| Zoom | 101 (18%) |
| Skype | 56 (10%) |
| Microsoft Teams | 53 (9%) |
| Bilateral Base | 17 (3%) |
| Google Meet | 6 (1%) |
| Attend Anywhere | 21 (4%) |
| VSee | 5 (1%) |
| GoTo meetings | 1 (0%) |
| WhatsApp | 33 (6%) |
| Facetime | 24 (4%) |
| Facebook Messenger | 2 (0%) |
| Telephone | 21 (4%) |
| Other | 18 (3%) |

Table 9 Extent troubled by issues of confidentiality, ethics and security

|  |  | Do you continue to be troubled |  |  | Row Total (%) |
| --- | --- | --- | --- | --- | --- |
|  |  | Not troubled at all | Somewhat troubled | Very troubled |  |
| Initially troubled | Not troubled at all | 215 | 0 | 0 | 215 (40%) |
|  | Somewhat | 183 | 112 | 0 | 299 (55%) |
|  | Very troubled | 11 | 21 | 6 | 38 (7%) |
| Column total (%) |  | 412 (75%) | 133 (24%) | 6 (1%) | 548 |

Table S10 Most preferred method

|  | Butterfly taps | 334 (59%) |
| --- | --- | --- |
|  | Online eye movements using a cursor on-screen | 25 (4%) |
|  | Online eye movements using a wand? | 17 (3%) |
|  | Online eye movements using screen-side visual dots at client end | 40 (7%) |
|  | Online eye movements using your own traditional arm movements | 36 (6%) |
|  | Client generated bilateral tones | 14 (2%) |
|  | Own EMDR-specific equipment | 15 (3%) |
|  | Hand-held buzzers | 4 (1%) |
|  | Other | 33 (6%) |
|  | Audio tones generated from your end | 19 (3%) |
|  | RemotEMDR | 16 (3%) |
|  | None given | 9 (2%) |
|  |  |  |

Table S11 Methods also used regularly

| Method | n (%) |
| --- | --- |
|  | (N=562) |
| Butterfly taps | 102 (18%) |
| Online eye movements using a cursor on-screen | 19 (3%) |
| Online eye movements using a wand | 22 (4%) |
| Online eye movements using screen-side visual dots | 40 (7%) |
| Online eye movements using your own traditional ar | 28 (5%) |
| Client generated bilateral tones | 11 (2%) |
| Own EMDR-specific equipment | 7 (1%) |
| Hand-held buzzers | 5 (1%) |
| Other | 34 (6%) |
| Audio tones generated from your end as therapist | 22 (4%) |
| RemotEMDR | 9 (2%) |

Table S12 Methods used occasionally

| Method | n (%) |
| --- | --- |
|  | (N=562) |
| Butterfly taps | 13 (2%) |
| Online eye movements using a cursor on-screen | 3 (1%) |
| Online eye movements using a wand | 5 (1%) |
| Online eye movements using screen-side visual dots | 4 (1%) |
| Online eye movements using your own traditional arm movements | 8 (1%) |
| Client generated bilateral tones | 6 (1%) |
| Own EMDR-specific equipment | 1 (0%) |
| Hand-held buzzers | 2 (0%) |
| Other | 4 (1%) |
| Audio tones generated from your end as therapist | 12 (2%) |
| RemotEMDR | 1 (0%) |

Table 13 Changes in Bilateral stimulation methods

|  |  | After |  |  |  |  |  |  |  | Row Total (%) |
| --- | --- | --- | --- | --- | --- | --- | --- | --- | --- | --- |
|  | | Bilateral tones | Butterfly taps | Cloud / RemotEMDR | Online eye movements (other) | screen-side dots  at client end | Online eye movements using therapist's own traditional arm movements | Other taps | Therapist's own equipment or not specified |  |
| Before | Butterfly taps | 4 (0.7%) | 0 (0.0%) | 8 (1.4%) | 13 (2.3%) | 19 (3.4%) | 4 (0.7%) | 5 (0.9%) | 5 (0.9%) | 58 (0.9%) |
|  | Client generated bilateral tones | 0 (0.0%) | 1 (0.2%) | 0 (0.0%) | 0 (0.0%) | 1 (0.2%) | 0 (0.0%) | 0 (0.0%) | 0 (0.0%) | 2 (0.0%) |
|  | Cloud / RemotEMDR | 0 (0.0%) | 0 (0.0%) | 1 (0.2%) | 0 (0.0%) | 0 (0.0%) | 0 (0.0%) | 0 (0.0%) | 0 (0.0%) | 1 (0.0%) |
|  | Hand-held buzzers | 0 (0.0%) | 7 (1.2%) | 0 (0.0%) | 0 (0.0%) | 1 (0.2%) | 1 (0.2%) | 0 (0.0%) | 0 (0.0%) | 9 (0.0%) |
|  | Online eye movements (other) | 0 (0.0%) | 14 (2.5%) | 2 (0.4%) | 2 (0.4%) | 1 (0.2%) | 5 (0.9%) | 0 (0.0%) | 1 (0.2%) | 25 (0.2%) |
|  | Online eye movements using therapist's own traditional arm movements | 1 (0.2%) | 18 (3.2%) | 5 (0.9%) | 4 (0.7%) | 6 (1.1%) | 0 (0.0%) | 0 (0.0%) | 1 (0.2%) | 35 (0.2%) |
| Column total |  | 6 (1.1%) | 46 (8.2%) | 19 (3.4%) | 19 (3.4%) | 28 (5.0%) | 10 (1.8%) | 5 (0.9%) | 35 (0.2%) | 140 (24.9%) |

Table 14 Effectiveness of bilateral stimulation methods used

| Method | Number using | How effective |  |  |
| --- | --- | --- | --- | --- |
|  | n (%) | very effective | partially effective | generally not very effective |
| Butterfly taps | 434 (77%) | 358 (82%) | 72 (17%) | 4 (1%) |
| Online eye movements using a cursor on-screen | 43 (8%) | 0 (0%) | 36 (84%) | 7 (16%) |
| Online eye movements using a wand | 39 (7%) | 32 (82%) | 6 (15%) | 1 (3%) |
| Online eye movements using screen-side visual dots | 79 (14%) | 68 (86%) | 8 (10%) | 3 (4%) |
| Online eye movements using your own traditional arm movements | 64 (11%) | 48 (75%) | 12 (19%) | 4 (6%) |
| Client generated bilateral tones | 25 (4%) | 21 (84%) | 3 (12%) | 1 (4%) |
| Own EMDR-specific equipment | 22 (4%) | 18 (82%) | 3 (14%) | 1 (5%) |
| Hand-held buzzers | 9 (2%) | 3 (33%) | 1 (11%) | 5 (56%) |
| Other | 66 (12%) | 0 (0%) | 58 (88%) | 8 (12%) |
| Audio tones generated from your end as therapist | 40 (7%) | 33 (83%) | 6 (15%) | 1 (3%) |
| RemotEMDR | 25 (4%) | 0 (0%) | 24 (96%) | 1 (4%) |

Table S15 Enhancing and Compromising Factors of online EMDR compared to in-person

| Extent enhanced / compromised | n (%) |
| --- | --- |
|  | (N=562) |
| Extremely enhanced | 95 (17%) |
| Somewhat enhanced | 183 (33%) |
| A mix of compromising and enhancing factors | 223 (40%) |
| Somewhat compromised | 44 (8%) |
| Extremely compromised | 13 (2%) |
|  |  |

Table S16 How well dealt with different aspects of therapy

| Situation | Responded | How effective |  |  |  |  |
| --- | --- | --- | --- | --- | --- | --- |
|  | n (%) | Extremely effectively | Very effectively | Moderately effectively | Slightly effectively | Not effectively at all |
| Building relationships with clients | 560 (100%) | 249 (44%) | 249 (44%) | 48 (9%) | 9 (2%) | 5 (1%) |
| Dealing with abreactions | 554 (99%) | 105 (19%) | 278 (50%) | 140 (25%) | 22 (4%) | 9 (2%) |
| Disassociation | 538 (96%) | 74 (14%) | 202 (38%) | 206 (38%) | 40 (7%) | 16 (3%) |
| Intense client affect | 556 (99%) | 120 (22%) | 288 (52%) | 111 (20%) | 33 (6%) | 4 (1%) |

Client results

Table S17 Client context

| Question | Response (only one) | n (%) |
| --- | --- | --- |
|  |  | (N=148) |
|  |  |  |
| Whether done in-person or online EMDR only or both | All my experience of EMDR therapy have been online | 84 (57%) |
|  | I have experiences of both in-person and online EMDR therapy, but they were separate programmes of therapy (either with the same person or a different therapist | 27 (18%) |
|  | I switched from in-person to online EMDR during a programme of therapy | 37 (25%) |
|  |  |  |
| Client's age group | 18 to 24 | 9 (6%) |
|  | 25 to 44 | 67 (45%) |
|  | 45 to 64 | 58 (39%) |
|  | 65 or older | 13 (9%) |
|  | Missing Data | 1 (1%) |
|  |  |  |
| Proportion of sessions online | <30% | 5 (3%) |
|  | 30% to 69% | 20 (14%) |
|  | 70% to 99% | 40 (27%) |
|  | 100% | 82 (55%) |
|  | Missing Data | 1 (1%) |
|  |  |  |
| Is your online EMDR therapy continuing or completed? | Completed | 68 (46%) |
|  | Continuing | 80 (54%) |
|  |  |  |

Table S18 Would you recommend online EMDR

| Question | Response (only one) | n (%) |
| --- | --- | --- |
|  |  | (N=148) |
|  |  |  |
| How enthusiastic would you be in recommending online EMDR | Cannot say at this point | 7 (5%) |
|  | Fairly enthusiastic | 22 (15%) |
|  | Not at all enthusiastic | 4 (3%) |
|  | Very enthusiastic | 115 (78%) |
|  |  |  |

Table S19 Enhancing and Compromising Factors of online EMDR compared to in-person

|  | Factor | n (%) |
| --- | --- | --- |
|  |  | (N=148) |
| Enhanced | Felt secure in my own environment e.g. home | 102 (69%) |
|  | Working via a screen helped me focus on the therapy | 32 (22%) |
|  | Appreciated not needing to travel | 100 (68%) |
|  | I was able to install/tap-in my special place and resources more efficiently | 20 (14%) |
|  | I felt more secure/more at ease in establishing rapport with my therapist | 28 (19%) |
|  | I felt this way of working to be more contained and focused | 31 (21%) |
|  | I found it easier to process my material online than I feel I would have in-pers | 21 (14%) |
|  | I found the closeness reassuring and relationally well connected. | 56 (38%) |
|  | Easier to co-create a supportive space. | 38 (26%) |
|  | Other | 28 (19%) |
|  |  |  |
| Compromise | Difficulty finding appropriate space in my home | 24 (16%) |
|  | Distractions in/near the space I was using | 50 (34%) |
|  | Privacy issues | 26 (18%) |
|  | Internet security issues | 9 (6%) |
|  | Difficulty expressing emotion | 21 (14%) |
|  | Difficulty interpreting body language | 30 (20%) |
|  | Technical incompatibility | 14 (9%) |
|  | Poor internet connection | 52 (35%) |
|  | Poor audio quality | 12 (8%) |
|  | Poor video quality | 14 (9%) |

Table S20 How views about online changed for those who experienced both

|  |  | Comfort with online now |  |  |  |  | Row Total (%) |
| --- | --- | --- | --- | --- | --- | --- | --- |
|  |  | Extremely | Somewhat | Neither | Somewhat | Extremely |  |
|  |  | comfortable | comfortable |  | uncomfortable | uncomfortable |  |
| how feel about switch from in-person? | Extremely apprehensive | 0 | 1 | 1 | 2 | 1 | 5 (8%) |
|  | Very apprehensive | 1 | 5 | 1 | 1 | 0 | 8 (13%) |
|  | Somewhat apprehensive | 17 | 10 | 4 | 3 | 0 | 34 (55%) |
|  | Not at all apprehensive | 14 | 1 | 0 | 0 | 0 | 15 (24%) |
| Column total (%) |  | 32 (52%) | 17 (27%) | 6 (10%) | 6 (10%) | 1 (2%) | 62 |

Table S21 Level of comfort with online EMDR now

| Comfort now | n (%) |
| --- | --- |
|  | (N=148) |
| Extremely comfortable | 94 (64%) |
| Somewhat comfortable | 36 (24%) |
| Neither comfortable nor uncomfortable | 9 (6%) |
| Somewhat uncomfortable | 8 (5%) |
| Extremely uncomfortable | 1 (1%) |

Table S22 Preference for online or in-person in future

| Degree of Preference | n (%) |
| --- | --- |
|  | (N=148) |
| Strong preference for online | 43 (29%) |
| Slight preference for online | 25 (17%) |
| No preference | 23 (16%) |
| Slight preference for in-person | 28 (19%) |
| Strong preference for in-person | 29 (20%) |

Table S23 Software platforms used

| Question | Response (only one) | n (%) |
| --- | --- | --- |
|  |  | (N=148) |
|  |  |  |
| On which online meeting platform did you and your therapist first meet? | Attend Anywhere | 2 (1%) |
|  | Bilateral Base | 21 (14%) |
|  | Microsoft Teams | 8 (5%) |
|  | Not sure | 5 (3%) |
|  | Other (please specify in the t | 3 (2%) |
|  | Skype | 10 (7%) |
|  | Telephone | 2 (1%) |
|  | WhatsApp | 2 (1%) |
|  | Zoom | 95 (64%) |
|  |  |  |
| Which online meeting platform did you and your therapist find yourself settling on? | Attend Anywhere | 2 (1%) |
|  | Bilateral Base | 33 (22%) |
|  | Microsoft Teams | 8 (5%) |
|  | Not sure | 6 (4%) |
|  | Other (please specify in the t | 1 (1%) |
|  | Skype | 7 (5%) |
|  | WhatsApp | 2 (1%) |
|  | Zoom | 89 (60%) |
|  |  |  |

Table S24 Method most commonly used by therapist

|  | Butterfly taps | 64 (43%) |
| --- | --- | --- |
|  | Online eye movements | 24 (16%) |
|  | Bilateral tones generated on y | 14 (9%) |
|  | Hand movements on-screen | 11 (7%) |
|  | Screen-side visual dots on you | 11 (7%) |
|  | Your own EMDR-specific equipment | 2 (1%) |
|  | Moving a pole or pointing stick | 2 (1%) |
|  | Hand-held buzzers | 2 (1%) |
|  | Other | 2 (1%) |
|  | Audio tones generated at the t | 7 (5%) |
|  | RemotEMDR | 1 (1%) |
|  | None given | 8 (5%) |
|  |  |  |

Table S25 Method also used regularly

| Method | n (%) |
| --- | --- |
|  | (N=148) |
| Butterfly taps | 1 (1%) |
| Online eye movements | 0 (0%) |
| Bilateral tones generated on your own machine | 1 (1%) |
| Hand movements on-screen | 2 (1%) |
| Screen-side visual dots on your own machine | 2 (1%) |
| Your own EMDR-specific equipment | 0 (0%) |
| Moving a pole or pointing stick | 2 (1%) |
| Hand-held buzzers | 2 (1%) |
| Other | 1 (1%) |
| Audio tones generated at the therapist end | 1 (1%) |
| RemotEMDR | 0 (0%) |

Table S26 Method used occasionally

| Method | n (%) |
| --- | --- |
|  | (N=148) |
| Butterfly taps | 22 (15%) |
| Online eye movements | 22 (15%) |
| Bilateral tones generated on your own machine | 5 (3%) |
| Hand movements on-screen | 17 (11%) |
| Screen-side visual dots on your own machine | 11 (7%) |
| Your own EMDR-specific equipment | 0 (0%) |
| Moving a pole or pointing stick | 1 (1%) |
| Hand-held buzzers | 0 (0%) |
| Other | 2 (1%) |
| Audio tones generated at the therapist end | 11 (7%) |
| RemotEMDR | 0 (0%) |

Table S27 How bilateral stimulation methods used

| Question | Response (only one) | n (%) |
| --- | --- | --- |
|  |  | (N=148) |
|  |  |  |
| Which of the below statements most accurately reflect | I generated, started and stopped | 8 (5%) |
|  | I have experienced a mix of BL | 27 (18%) |
|  | I was guided to tap my own bod | 59 (40%) |
|  | My therapist generated and sta | 49 (33%) |
|  | Missing Data | 5 (3%) |
|  |  |  |

Table S28 Effectiveness of different aspects of therapy

| Aspect | Responded | How effectively used |  |  |  |  |
| --- | --- | --- | --- | --- | --- | --- |
|  | n (%) | Extremely effectively | Very effectively | Moderately effectively | Slightly effectively | Not effectively at all |
| Bilateral Stimulation | 144 (97%) | 66 (46%) | 45 (31%) | 24 (17%) | 7 (5%) | 2 (1%) |
| Installation of a safe/special place and resources | 132 (89%) | 48 (36%) | 40 (30%) | 32 (24%) | 9 (7%) | 3 (2%) |
| Focus on specific memory targets | 136 (92%) | 56 (41%) | 0 (0%) | 19 (14%) | 55 (40%) | 6 (4%) |
| Identifying negative thoughts, emotions and body sensations before sets of BLS | 137 (93%) | 51 (37%) | 0 (0%) | 21 (15%) | 60 (44%) | 5 (4%) |
| Identifying an alternative positive cognition/thought before starting the process | 133 (90%) | 36 (27%) | 60 (45%) | 25 (19%) | 11 (8%) | 1 (1%) |
| Use of numerical scales to check progress (Subjective Units of Disturbance (SUD) | 129 (87%) | 33 (26%) | 46 (36%) | 26 (20%) | 13 (10%) | 11 (9%) |
| Tight session structure with regular appropriate returns to the target memory | 133 (90%) | 42 (32%) | 56 (42%) | 23 (17%) | 10 (8%) | 2 (2%) |

**Evaluation of the delivery of online EMDR**

Interview Guide

1. Warm-up– let’s start out with a very general question. Separate from the challenge of the Covid pandemic and lockdowns themselves, have you enjoyed being an EMDR therapist during this extraordinary time?

1. Did you initially have any discomfort about delivering EMDR online?

Prompt: Please can you tell me more about that?

1. Are you comfortable delivering EMDR online now?

Prompt: Please can you tell me more about why you feel comfortable with it?

1. Do you feel safe working online?

1. Do you feel comfortable about client confidentiality when working online?

1. How enthusiastic are you about providing EMDR online in the future?

Prompt: What would you say to therapists who were worried about using EMDR online?

1. In your survey answers, you highlighted [*refer to one or two options ticked in survey question 4.1 - or flagged in 4.2*] as factors that enhanced your experience of delivering EMDR online services as against in-person delivery. Please could you elaborate on that?

1. In your survey answers, you highlighted [*refer to one or two options ticked in survey question 4.3 - or flagged in 4.4*] as factors that compromised your experience of delivering EMDR online services as against in-person delivery. Please could you elaborate on that?

1. In your survey responses you mentioned some adaptations you’ve made to the delivery of EMDR when working online [*refer to answers to 5.2, 5.3*]. Could you tell me about these?

Prompt: [*Follow up if participant has answered they feel online working compromised their work in survey question 6.1*]?

1. Bilateral stimulation is a core component of EMDR. Please can you tell me about your experience of working with different bilateral stimulation techniques online?

Prompt: Please can you tell me more about why you found particular techniques effective / ineffective [*Recite back answers from survey 5.12*]?

1. [*Optional - depending on answers to survey section 6*] You indicated that you moved clients between online EMDR and in-person / group [*or vice versa*]. Please could you say more about why and how this happened?

1. Is there anything else you think we should have covered in this research?

That concludes the interview. Thank you.
